# The Role of Arp2/3 in End‐Resection During DNA Double‐Strand Break Repair

**DOI:** 10.1002/bies.70171

**Published:** 2026-09-09

**Authors:** Felix Y. Zhou, James E. Haber

**Affiliations:** ^1^ Rosenstiel Center and Department of Biology Brandeis University Waltham Massachusetts USA; ^2^ Radiation Oncology Department Dana Farber Cancer Institute Boston Massachusetts USA

**Keywords:** biology, cell biology, chromatin, DNA, DNA damage, DNA repair, homologous recombination, Rad51

## Abstract

The efficient repair of chromosome breaks by homologous recombination depends on the ability of the ends of a double‐strand break (DSB) to locate homologous sequences to serve as templates to repair the lesion. These repair mechanisms are conserved across eukaryotic evolution. Repair depends on 5’ to 3’ resection of the broken ends, allowing the assembly of a Rad51 filament. In yeast, fruit flies, and mammals, broken chromosome ends display increased mobility, allowing the ends to explore a larger volume. The Arp2/3 actin nucleator complex plays a key role in increased local chromatin mobility near a DSB. In budding yeast, Arp2/3, LAS17^WASP^, and myosins are required both to initiate and to maintain long‐range end resection, affecting both DNA repair and the maintenance of the DNA damage checkpoint. At least in budding yeast, the connection between controlling long‐range resection and increased mobility is direct, as inhibiting resection prevents damage‐induced chromatin mobility. Moreover, although the search for homology by Rad51‐coated broken DNA ends would seem to be improved by greater exploration of the nuclear volume, increased local mobility through Arp2/3 per se does not affect the efficiency of ectopic recombination in yeast. Here, we review the recently defined roles of nuclear actin in DNA damage repair, with a focus on new observations in budding yeast.

## Graphical abstract

Arp2/3 has an evolutionarily conserved role in promoting 5’ to 3’ resection of DSBs, RAD51 filament formation, maintenance of the DNA damage checkpoint, increased mobility of DSBs, and relocation of damage in heterochromatin regions to the nuclear periphery. This activity is mediated by myosin proteins and Las17_WASP_ which additionally facilities RPA loading onto ssDNA.

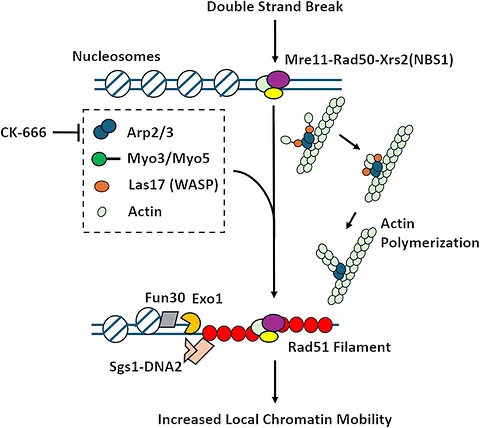

## 1 Introduction

**FIGURE 1:**
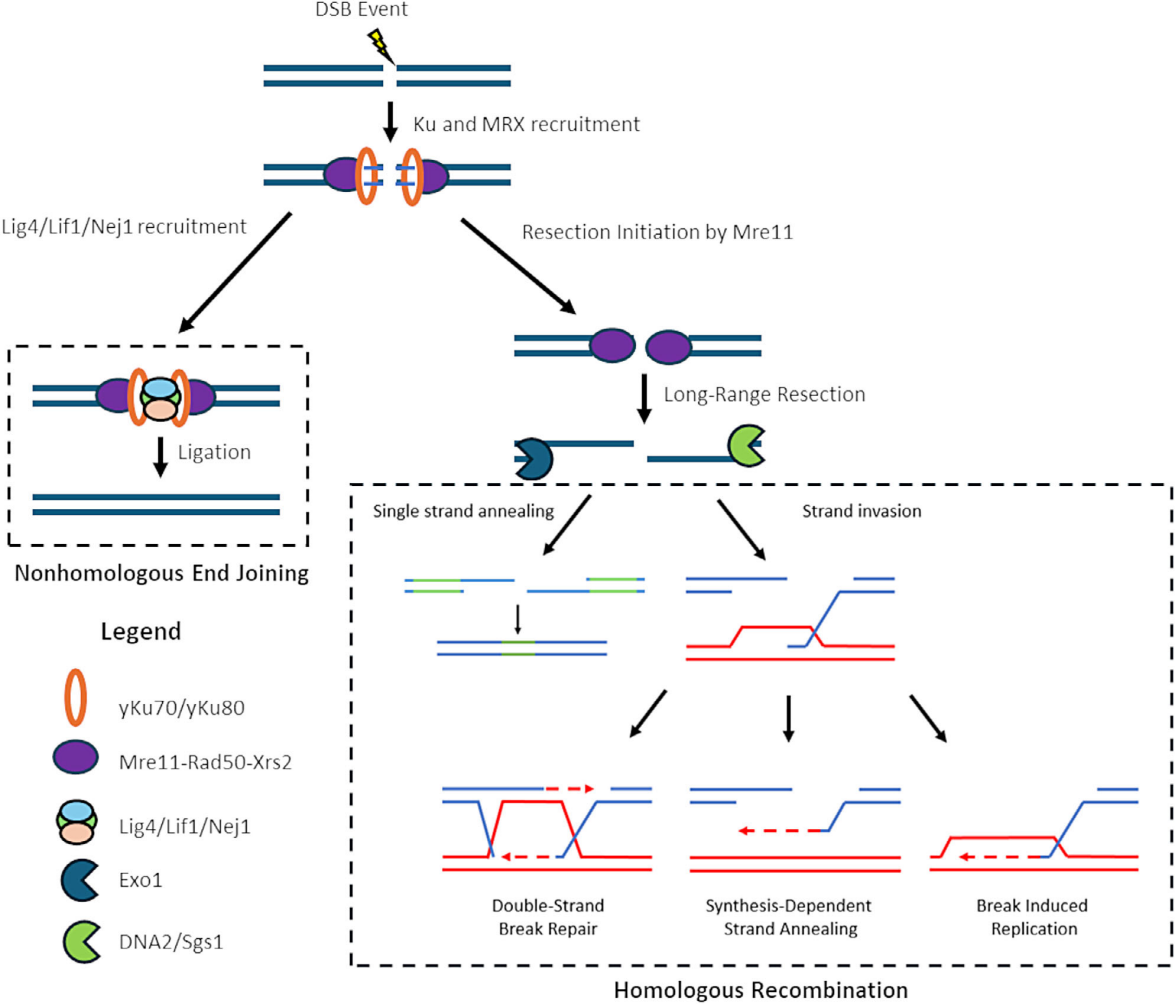
DSB repair pathways and end resection. Following a DSB event, MRX^MRN^ and yKu70/80 are rapidly recruited to the ends of the DSB. Repair by nonhomologous end joining (NHEJ) ligates the ends of the DSB together. Resection through Mre11 initiates repair through homologous recombination (HR). The ends of the DSB are resected in a 5’ to 3’ manner. If there is shared homology flanking both sides of the DSB, then the DSB can be repaired through single strand annealing (SSA). Otherwise, once a region of homology is found, one end of the DSB will invade the dsDNA to form a D‐loop. Synthesis can then proceed either through DSB repair where the other end of the DSB will engage the D‐loop, by synthesis‐dependent strand annealing where the newly synthesized strand dissociates from the donor to anneal to the other end of the DSB, or through break‐induced replication where synthesis continues to the end of the telomere.

Efficient repair of double strand breaks (DSBs) by homologous recombination (HR) is critical in maintaining genome stability. A key early step in repair is the creation of 3’‐ended single‐stranded DNA by end‐resection so that the Rad51 recombinase can form a filament that can initiate a search for a homologous donor template that can be copied to patch up the lesion (Figure 1). End‐resection also triggers activation of the DNA damage checkpoint, mediated by the Mec1^ATR^ kinase (reviewed in the literature [1]) (throughout this review we will discuss Saccharomyces *cerevisiae* proteins but indicate their human homologs as superscripts). The initial resection of DSB ends relies on the Mre11‐Rad50‐Xrs2^NBS1^ complex, which is rapidly recruited to the DSB (reviewed in Ref. [2]). Mre11's 5’ and 3’ endonuclease and exonuclease activities play a role both in end processing and removing protein adducts from DSB ends [3, 4, 5]. MRX removes the terminal 5’‐ended segments of broken ends, resulting in short (≤100 nt) 3’‐ended ssDNA ends. Much more extensive end‐resection is carried out by two redundant, but different exonucleases: by the helicase‐endonuclease Sgs1‐Rmi1‐Top3‐Dna2 complex (BLM‐RMI1‐RMI2‐Top3α‐DNA2 in mammals) and by Exo1 [6, 7]. In addition, long‐range resection depends on the activity of the chromatin remodeler Fun30^SMARCAD1^ [8, 9]. The Mre11 complex also recruits the kinase, Tel1^A^, to the break; but Tel1 plays a minor role both in repair and in establishing and maintaining the DNA damage response, which in yeast blocks cell cycle progression at G_2_/M, prior to mitosis. In this review, we cover recent findings on the role of actin nucleation on DSB mobility, end resection, and activation of the DNA damage checkpoint as well as highlight key areas of interest for future studies.

### 1.1 Loading of Rad51 and the Search for Homology

In HR, the ends of the DSB are resected to allow the loading of the recombinase protein Rad51 onto ssDNA. The newly resected 3’‐ended ssDNA is rapidly coated with replication protein A (RPA) to prevent secondary structure formation, protect the DNA from nucleases, and recruit the checkpoint kinase, Mec1‐Ddc2 [10, 11]. Rad52 displaces RPA and mediates the loading of the Rad51 onto ssDNA to create a Rad51 filament [12, 13, 14]. The Rad51 filament is required for homology search and most forms of repair by HR [15]. When Rad51 is loaded it forms a highly structured nucleoprotein filament with each copy of Rad51 occupying three bases of DNA, stretching the DNA 1.5 times [16, 17].

The search for homology by the Rad51 filament remains poorly understood. Intrachromosomal searches are more efficient than interchromosomal [18, 19, 20]. Some evidence suggests that the yeast Rad51 filament can diffuse and contact DNA as a function of the proximity of sequences within the nucleus (contact probability) as well as to “slide” along the chromosome [18, 19, 21, 22]. It seems that the two ends move together: the coordinate action of both ends has been supported by genetic and molecular biological analysis of DSB repair involving both gene conversion and single‐strand annealing [23].

### 1.2 DSBs Induce Increases in Both Local and Global Chromosome Movement

**FIGURE 2:**
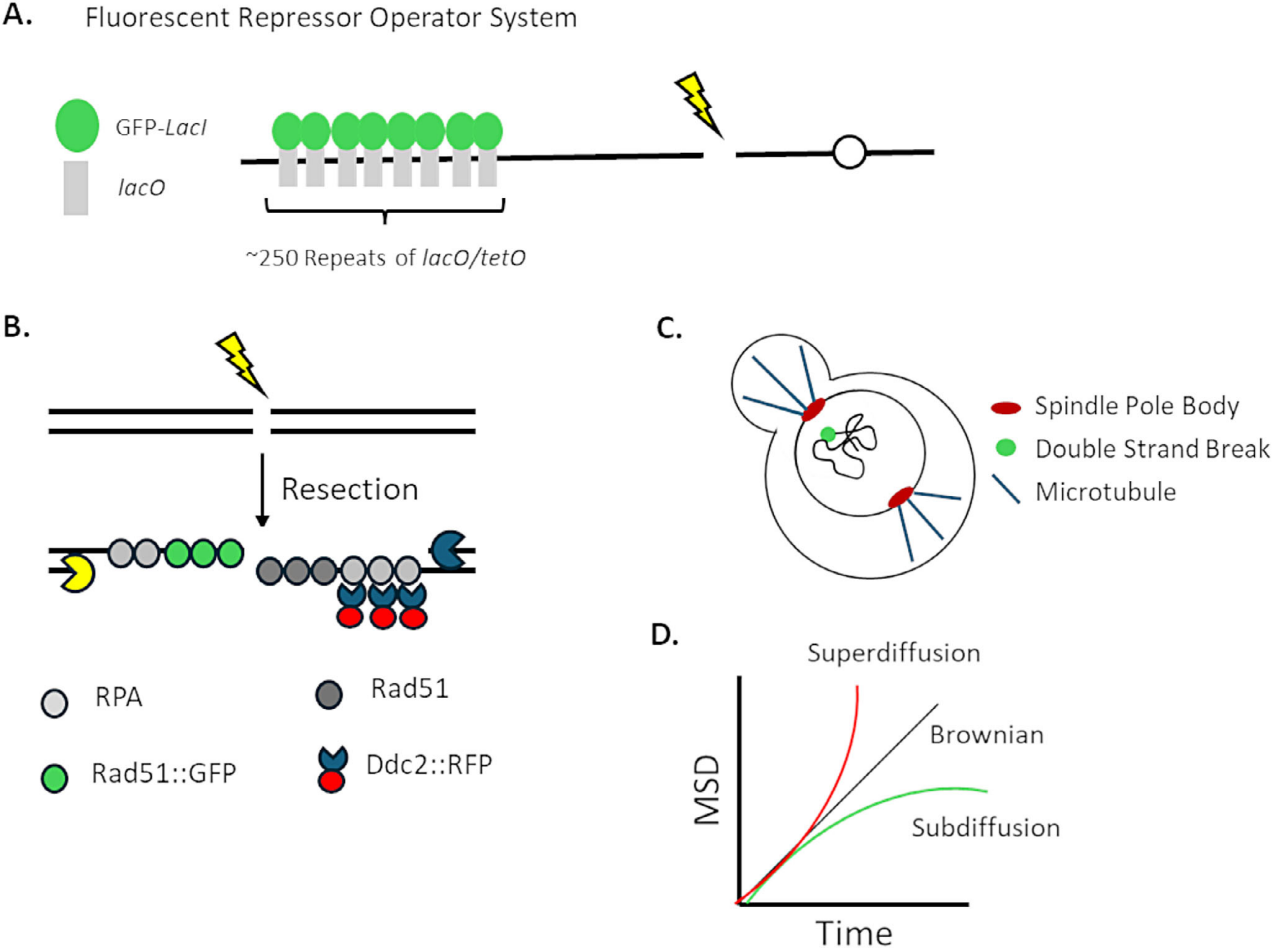
Models for labeling and tracking of DSBs. (A) Model of the fluorescent repressor operator system (FROS) for labeling chromatin through expression of a fluorescently tagged repressor protein (GFP‐*LacI* or GFP*‐TetO*) and insertion of a *lacO* or *tetO* array (typically 250 repeats) into the chromatin either near an inducible DSB site or on an undamaged chromosome to measure changes in DSB mobility or global changes in chromatin mobility respectively. (B) Model for direct labeling of DSBs using fluorescently tagged DSB repair proteins that are recruited directly to resected, broken chromosome ends. (C) Model of single‐particle tracking (SPT) of a GFP focus and the path of the DSB over time, relative to a fiduciary mark, such as the yeast spindle pole bodies (SPBs). (D) Example of a mean squared displacement (MSD) curve.

Pioneering studies by the laboratories of both Susan Gasser and Rodney Rothstein demonstrated that single DSBs in budding yeast resulted in significant changes in chromatin mobility within the nucleus [24, 25]. DNA damage‐induced changes in chromosome mobility in yeast have been assessed both by treatment with radiomimetic drugs such as zeocin or by the induction of site‐specific DSBs by HO and I‐SceI endonucleases [24, 25, 26, 27]. In metazoans, most work has been performed using ionizing radiation and endonucleases such as I‐SceI. The broken ends exhibit a significantly increased exploration of the nuclear volume, as measured by monitoring the movements of a fluorescent mark adjacent to a DSB or a fluorescent protein that binds in multiple copies to resected DNA ends (Ddc2^ATRIP^‐GFP or Rad51‐GFP) relative to the nuclear periphery or the spindle pole bodies (Figure 2A,B) [24, 25, 27, 28, 29]. In budding yeast, a single DSB causes both a “local” increase in chromatin mobility near a DSB and a general “global” increase of undamaged chromosomes [24, 25, 30]. Global changes in chromatin mobility are not essential for repair of DSBs near centromeres, but they become important for the repair of DSBs outside the centromere through HR [30].

Changes in DSB mobility were measured using mean squared displacement (MSD) analysis (reviewed in Refs. [31, 32, 33]). In short, the 3D position of a particle is tracked over time, *t*. MSD analysis provides the mode of movement (α), the maximal diffusion coefficient (*D*_i_), and the radius of confinement (*R*_C_). On a log‐log plot of lag‐time (*τ*) versus MSD yields a line with slope *α* (Figure 2C). Lag‐time, *τ*, is the specific time interval over which a particle's movement is measured. The *α* describes the type of diffusion the particle undergoes: *α* = 1 exhibits Brownian motion (normal diffusion), *α* ≠ 1 reflects anomalous diffusion, *α* > 1 shows directed motion (superdiffusion), and *α* < 1 shows confined motion (subdiffusive). When a particle exhibits confined motion, the slope of the MSD curve will plateau, and the *R*_C_ can be calculated to define the volume that a particle explores. Studies have shown that chromatin movement is not governed by simple Brownian motion and that ATP is required for motion (reviewed in Refs. [31, 34]).

DNA damage‐induced increased mobility in yeast is tied to the activation of the DNA damage checkpoint. Mobility depends on activation of Mec1^ATR^ kinase and on its phosphorylation of targets such as Rad9 [24, 35]. One modification that defines the region around the DSB is the phosphorylation of histone protein H2A^H2AX^. Phosphorylation of H2A‐S129 extends about 50 kb on either side of the DSB in yeast [36]. The importance of this modification is evident from the observation that chromosome mobility of undamaged chromatin is increased when a phosphomimetic H2A‐S129E replaces H2A [37]. However, although *mec1*Δ cells fail to display an increased mobility around the DSB, the H2A histone proteins in the region around the DSB are still phosphorylated, carried out by the related Tel1^A^ kinase; so the connection between γ‐H2AX and mobility is not yet well understood.

In yeast, DSB‐associated mobility also requires proteins involved in HR such as Rad51 and Sae2 [25]. One proposed model for how a DSB induces increased “local” chromatin mobility suggests that the recruitment of repair proteins to DSB sites causes liquid–liquid phase separation and allowing the DSB to move more freely through the nucleus [31]. Phase separation forms membraneless compartments that isolates chromatin regions. In *Drosophila*, Nup98 forms a condensate around DSB sites–that are immiscible with HP1 condensates–and relocates them to the nuclear periphery before Rad51 is loaded to ensure safe repair by HR [38]. DSB relocation to the nuclear periphery is discussed in further detail later. Rad52 is known to cause liquid–liquid phase separation and Rad52 droplets are shown to fuse to form “repair center” droplets through DNA‐damage‐induced intranuclear microtubule filaments (pti‐DIMs) and short aster‐like DIMs interact with longer DIMS to drive these repair centers to the nuclear periphery for repair [39, 40]. However, the presence of multiple DSBs does not always lead to the consolidation of DSBs into “repair centers,” as was shown in a 3‐DSB system in yeast [29, 41].

In *Drosophila* and in mouse cells, directed motion of DSBs (and possibly other damage) is seen particularly for damage in heterochromatic regions, where moving the damaged DNA away from the surrounding highly repeated DNA sequences prevents recombinational events that would lead to chromosome rearrangements [42, 43]. In yeast, this relocation is seen when ribosomal DNA is damaged (i.e., in repeated DNA sequences) [44], when DSBs are left unrepaired for a long time [45, 46, 47], or at stalled replication forks [48]. In *Drosophila*, damage in heterochromatin regions is relocated to the nuclear periphery in an actin‐ and myosin‐dependent manner [49, 50]. Why there is no equivalent motion of damage in euchromatin can be rationalized as unnecessary in the absence of surrounding repeated sequences.

### 1.3 Actin Nucleation by the Arp2/3 Complex

A role for actin in chromosome dynamics was first reported in budding yeast [51] and nuclear F‐actin has now been shown in flies and mammalian cells [43, 52]. Actin is an essential 42‐kDa protein that is well conserved across evolution that can exist as monomers (G‐actin) or in filaments (F‐actin) [53, 54]. Polymerization of G‐actin into long non‐branching filaments is promoted by formins [55] and polymerization into branching actin networks is promoted by the Arp2/3 complex, hereby referred to as Arp2/3 [56, 57, 58]. Actin filaments are core components of the cytoskeleton and facilitate many essential functions such as cell motility, providing mechanical motion, and cargo transport (reviewed in Ref. [59]). In simple eukaryotes, such as budding or fission yeasts, these different actin functions are carried out with only a single actin gene [60]. However, higher organisms carry multiple isoforms of actin that are categorized based on their function: skeletal muscle α‐actin, smooth muscle α‐actin (vascular), cardiac muscle α‐actin, smooth muscle γ‐actin (enteric), and non‐muscular β‐actin and γ‐actin (reviewed in Ref. [59]).

Myosins are a family of motor proteins that work together with actin structures to carry out a variety of functions such as cargo transport, cell motility, and muscle contraction (reviewed in Ref. [61]). Budding yeast contains five myosin proteins (reviewed in Ref. [62]), of which two (Myo3 and Myo5) have a canonical role in clathrin‐mediated endocytosis and a newly characterized role in double‐strand break (DSB) mobility [28, 63].

Arp2/3 is evolutionarily conserved [64] and in eukaryotes is known to form branching actin networks at the cell membrane to facilitate cell motility and endocytosis [57, 58]. Actin nucleation through Arp2/3 was first characterized through its role in the propulsion of the pathogenic bacterium *Listeria monocytogenes* [65]. From purified *L. monocytogenes* cell extracts, they found an eight‐protein complex that was sufficient for making actin filaments with ActA [65]. These eight proteins were composed of the seven‐protein complex Arp2/3 and a nucleation promoting factor (NPF), later identified as Wiskott–Aldrich Syndrome protein (WASP). WASP was first identified in Wiskott–Aldrich Syndrome patients which is a rare X‐linked recessive disorder which is linked to dysregulation of the actin cytoskeleton in hematopoietic cells [66]. In humans, the WASP family of proteins contains nine different members which are divided into five groups of proteins based on sequence similarity: WASP, SCAR/WAVE, WASH, WHIMP, and JMY (reviewed in Ref. [67]).

WASP stimulates Arp2/3 actin polymerization in human cells through its VCA (Verprolin homology, Cofilin homology, Acidic region) domain at the C‐terminal end [68]. The CA domain interacts with Arp2/3 to cause Arp2/3 to undergo a conformational change to a “closed” state that mimics an actin dimer and the V domain brings G‐actin to Arp2/3 [68]. This activity is inhibited by the drug CK‐666 [69]. Las17 is the primary WASP‐like protein found in budding yeast with additional nucleation promoting activity from verprolin (Vrp1) and the type‐I myosins Myo3 and Myo5 [70, 71, 72, 73]. Arp2/3 is essential for the initiation and facilitation of resection of broken DNA ends in budding yeast and human cells [28, 52]. This function affects other DNA damage repair processes such as the DNA damage checkpoint (DDC) and repair through HR.

There is substantial evidence of additional roles played by actin within the eukaryotic nucleus. In mammalian oocytes, nuclear actin filaments have been shown to play a role in assuring proper chromosome segregation at the first meiotic division [74, 75]. In all eukaryotes, a set of actin‐related proteins are known to be part of chromatin remodelers, such as INO80, SWR1, and NuA4 [76]. In some studies, G‐actin itself is also found in these complexes [77]. In humans, β‐actin has a direct role in transcription through the recruitment of all three classes of RNA polymerases, regulating the subcellular localization of transcription factors, and binding to mRNPs (reviewed in Ref. [76]). Coimmunoprecipitation experiments have shown that actin binds to all three classes of RNA polymerases found in eukaryotes [78, 79, 80]. Association with β‐actin is required for RNA polymerase activity, as depletion of free β‐actin through β‐actin antibodies impaired RNA polymerase activity both in vitro and in vivo [78, 79, 80]. One proposed mechanism for the role of actin in transcription regulation is that the nuclear localization of transcription factors is affected by binding to G‐actin [81].

### 1.4 Arp2/3 And Double‐Strand Break Repair

The role of Arp2/3 in DSB repair was first characterized for its role in DSB mobility in U2OS and *Drosophila* cells [43, 52] and was later confirmed to be evolutionarily conserved in budding yeast [28]. A proteomics study in *Xenopus* egg extracts showed that all the components of Arp2/3 and β‐actin are enriched in chromatin containing DSBs and that this association was enhanced by the activities of the PI3‐like kinases ATM and ATR [52]. In U2OS cells, Arp2/3 and WASP were both found enriched at some AsiSI‐induced DSBs and nuclear actin was found to cluster at DSBs labeled with RPA32‐mCherry [52]. MSD analysis showed that the mobility of Rad52‐mCherry and RPA32–pEGFP–NLS foci was lower when cells were treated with the Arp2/3 inhibitor CK‐666 or when a conditional *Arp2c* allele was depleted [52]. In *Drosophila*, it was reported that the relocalization of DSBs in heterochromatin to the nuclear periphery was dependent on directed motion through Arp2/3 and the myosins MyoV, Myo1A, and Myo1B [43]. Depletion of the WASP family proteins Scar and Wash also caused a defect in relocalization of heterochromatic DSBs to the nuclear periphery [43]. The Smc5/6 complex, which is important for DSB relocalization to the nuclear periphery [44], was required for recruitment of the myosin activator Unc45 [43]. Inhibition of Unc45 had a similar negative effect on DSB relocalization [43]. Arp2/3 also contributes to the clustering of DNA damage foci in euchromatin in *Drosophila* and mammalian cells [43, 52].

The role of Arp2/3 in DSB mobility is conserved in budding yeast [28]. The nucleation promoting factors Las17^WASP^ and the class I myosins, Myo3 and Myo5, which are required for Arp2/3 nucleation during endocytosis, are required for increased DSB mobility at a site‐specific DSB [28]. Although Arp2/3 is required for endocytosis in budding yeast [72], it was shown that a deletion of *SLA2*, which blocks endocytosis, did not affect DSB mobility [28]. Taken together, the data from these studies points to an evolutionarily conserved role for Arp2/3 and myosin proteins in chromosome mobility induced by DNA damage.

### 1.5 Arp2/3 Initiates and Mediates End Resection in DNA Damage Repair

In addition to changes in chromosome mobility, Arp2/3 has been also implicated in the 5’ to 3’ processing of DSB ends. In U2OS cells, the size of RPA foci at radiation‐induced DNA damage was reduced by inhibiting Arp2/3 with CK‐666 and less ssDNA was detected at AsiSI site‐specific DSBs measured by qPCR analysis, suggesting a defect in generating single‐stranded DNA at DSB ends [52], although there could also be a reduction in RPA recruitment [82]. More direct measurements of DSB end‐processing in yeast have implicated Arp2/3 both in the initiation and the maintenance of long‐range resection [28].

Resection in budding yeast was monitored by a qPCR‐based resection assay surrounding a DSB site induced by the site‐specific HO endonuclease [28, 83, 84, 85]. When Arp2/3 activity was blocked before DSB induction, either by CK‐666 addition or auxin‐induced degradation of Las17, resection around the DSB was significantly impaired [28]. Resection was also reduced when Arp2/3 or Las17 was inactivated an hour after the break. Similar results were obtained by monitoring the exonuclease‐mediated loss of a LacI‐GFP focus bound to LacO sequences located 4 kb away from a DSB. These results suggest that Arp2/3 is needed both to initiate and to maintain long‐range resection [28].

The difference between initiating long‐range resection and maintaining it could be seen by over‐expressing Exo1. When CK‐666 was added prior both to DSB induction and overexpression of Exo1, resection was impaired despite Exo1 overexpression. However, when CK‐666 was added after Exo1 overexpression and DSB induction, the rate of resection was not impaired [28]. In G_1_‐arrested yeast cells, yKu70/80 acts as a barrier for long‐range resection [86], and deletion of the Ku70/Ku80 proteins allows Exo1 to degrade DSB ends [2, 87]. However, when yKu70 was deleted in cycling cells, it did not overcome the block to resection created by inhibiting Arp2/3 [28]. These results suggest Arp2/3 does not act by facilitating the removal of Ku proteins from DSB ends.

### 1.6 Myosins and DSB Mobility

Recent evidence has shown that myosins have a role in DNA damage repair. This is seen by changes in DSB mobility when either one of the class I myosins, Myo3 or Myo5, are deleted in budding yeast [28] and how the depletion of the Myo1a, Myo1b, or MyoV in *Drosophila* increases sensitivity to ionizing radiation, heterochromatin repair defects, and heterochromatin‐driven genome rearrangements [43]. Resection analysis shows that a single deletion of either *myo3* or *myo5* did not result in a significant change in resection but was sufficient to lower the mobility of the DSB [28]. A double deletion of *myo3* and *myo5* is known to be synthetically lethal and to disrupt nuclear architecture [88]. When the AID system was used to conditionally deplete both type‐I myosins, the rate of resection was disrupted, but the mobility of the DSB was found to return back to wildtype levels. This increase in DSB mobility might be due to a change in overall chromatin organization in the cell. New evidence shows that when both Myo3 and Myo5 are evicted from the nucleus it caused a massive change in chromatin organization [89]. In *Drosophila* and mammalian cells, appending a nuclear localization signal to a polymerization‐defective actin mutant (R62D) poisoned the damage‐induced chromosome movement required for HR repair [43, 52]; but such a strategy was unsuccessful in budding yeast (S. Gasser, personal communication, 2025). Depletion of Arp2/3, Las17^WASP^, or type‐I myosins from the nucleus [89] could be used to differentiate between the nuclear and cytoplasmic functions of these proteins during DSB repair in budding yeast.

### 1.7 Resection and Relocation of DSBs to the Nuclear Periphery/Pore

Precise control of DSB repair in heterochromatin regions containing repeated DNA sequences is required to prevent ectopic exchanges and maintain genome stability. Across evolution it has been shown that DSBs in heterochromatic regions are relocated to the nuclear periphery to be repaired through HR [43, 50, 90]. The stage of the cell cycle influences whether a DSB will be relocated to the nuclear periphery. In budding yeast, the interaction between a persistent DSB–such as Gal‐HO DSBs in the *MAT* locus with both donor sequences deleted [91]–with nuclear pore happened independently of cell cycle phase, but Msp3–an inner nuclear membrane SUN domain protein–binding to DSBs was S and G_2_ phase specific [92]. In mouse cells, DSBs created in G_1_ using CRISPR/CAS9 in major satellite repeats in pericentric heterochromatin regions prompted repair through NHEJ and did not require relocation [93]. However, when CRISPR/Cas9 made DSBs in the same locations in S/G_2_ phases, the DSBs were relocated to the periphery of the heterochromatin domain followed by recruitment of Rad51 [93]. In human and mouse cells, dambge‐induced phosphorylation of H2AX occurs in heterochromatin and then chromatin is expelled to the periphery of the heterochromatin in an ATM‐independent manner [94].

Initial steps of DSB repair, such as DSB detection and resection, occur in heterochromatin [50, 90, 93, 94, 95, 96] and relocation to the nuclear periphery requires resection. In budding yeast, extensive resection seems to be correlated with relocalization as observed when DSBs could be repaired through single strand annealing (SSA) (Figure 1). When the pair of homologous sequences flanking a DSB were 5 kb away from each other, the DSB was able to be repaired through SSA without relocation [45]. However, when the donors were 30 kb apart the DSB was found to be relocated to the nuclear periphery where it interacted with the nuclear envelope protein Mps3 [45].

The loading of Rad51 onto ssDNA is delayed in heterochromatin and rDNA regions by SUMOylation [44, 90]. In *Drosophila* and mice, loading of Rad51 occurs once DSBs have left heterochromatin or rDNA regions [44, 90, 93]. Again, it should be noted that the behavior of DSBs in heterochromatin is distinctly different from breaks in euchromatic DNA, where such direct movement to the periphery is not observed. In *Drosophila* this process is carried out by the SUMO E3 ligases dPIAS and the Smc5/6 subunits Nse2/Qjt (Quijote) and Nse2/Cerv (Cervantes) [49, 50, 96]. Relocation to the nuclear pore complex (NPC) might trigger desumoylation through the NPC‐associated protein Ulp1 and allow for Rad51 loading [48, 97]. In *Drosophila* relocation to the NPC and the inner nuclear membrane proteins (INMPs) occurs through the Smc5/6 associating proteins STUbL/RENi [50]. In budding yeast, repair of DSBs in rDNA or persistent DSBs requires relocation out of the nucleolus before the recruitment of recombination machinery [44, 92]. This relocation is dependent on Mre11, Smc5/6, and SUMOylation of Rad52; and loss of any of these proteins results in Rad52 focus formation in the nucleolus and rDNA hyperrecombination [44]. In budding yeast, SUMOylation of Rad52, Rad59, and all three subunits of RPA, Rfa1‐3, was required for relocation of collapsed forks to the nuclear periphery [48].

In *Drosophila*, it was shown that relocation of DSBs to the nuclear periphery was dependent on Arp2/3, nuclear myosins, Unc45, and Smc5/6 [43]. Recently, it has been shown that Nup98 forms condensates at DSBs before relocalization to the nuclear periphery through Sec13 and Nup88 [38]. Nup98 condensate formation occurs downstream of Smc5/6 recruitment and HP1 condensate formation [38]. Arp2/3 recruitment to DSBs in *Drosophila* was dependent on Mre11 as well as the heterochromatin protein HP1a and recruitment of the myosin activator Unc45 was dependent on Smc5/6 [43]. It is worth studying whether Arp2/3 and the type‐I myosins Myo3 and Myo5 have a conserved role in DSB relocation to the nuclear periphery and whether Arp2/3 regulated resection is dependent on this relocation in budding yeast. Since repair by SSA did not require relocation to the nuclear periphery when the homologous pairs were <5 kb apart, it is possible that inhibition of Arp2/3 will not affect repair by SSA under these conditions.

### 1.8 DSB End Resection and DSB Mobility

Given that a DSB causes a global increase in chromatin mobility [24, 25], it would seem the increase in exploration of the nuclear volume should facilitate homology search (reviewed in Refs. [32, 98, 99]). However, the relationship between changes in chromatin mobility and repair by HR is still not well defined. Histone degradation in response to DSBs increases chromatin mobility and recombination rates [100]. The position of a DSB relative to the centromere affected whether an increase in global chromatin mobility affected the importance of DSB‐induced chromatin mobility on repair efficiency through HR [30].

Previously, we showed that the efficiency of recombination between an HO endonuclease‐cleaved sequence and a donor located at sites on different chromosomes reflected their proximity [18, 19]. Deletion of Myo5 prevents damage‐induced increase in Rc, although it does not affect the rate of resection of DSB ends; but the efficiency of ectopic recombination was surprisingly unaltered [28], such that far‐away donors were not further impaired in their usage. Therefore, increased movement per se does not seem to be a central element in DSB repair. However, in this same system, when Las17‐AID was inactivated there was a significant inhibition of ectopic recombination of both nearby and distant donors [28]. The difference in the phenotypes of *myo5*Δ and Las17‐AID inactivation appears to reflect the fact that inactivating Las17 impairs the rate of end‐resection and the persistence of the DNA damage checkpoint. Loss of Las17 results in a much shorter G_2_/M arrest. However, when Las17‐inactivated cells were held in G_2_/M by addition of the microtubule polymerization inhibitor, nocodazole, repair was restored to wildtype levels [28]. These differences suggest that the defects in DSB repair are related to the persistence of the DNA damage checkpoint.

Since inactivation of Arp2/3 impaired both broken chromosome mobility and end‐resection, this prompted the question whether direct inhibition of resection would affect broken chromosome mobility. Indeed, when the redundant long‐range exonucleases Exo1 and DNA2/Sgs1 are both inactivated, the Rc of the broken chromosome ends was also reduced [28]. A similar result was found when the chromatin remodeler, Fun30, was deleted. Deleting Fun30 lowered the rate of resection from 4 kb/h to 1.2 kb/h [9] and prevented the normal increase in Rc that accompanies a DSB [28]. However, deletion of Fun30 increased repair efficiency through HR despite the lower DSB mobility [19, 28]. Interestingly, a deletion of Sae2 caused a delay in the initiation of resection which also delayed DSB‐induced chromatin mobility; however, once long range resection was triggered, there was no difference in DSB mobility between the wildtype and the *sae2* deletion mutant [25].

These data suggest that Arp2/3 plays a key role in facilitating long‐range resection and that the block in resection is what impairs the damage‐induced increase in chromosome mobility. HR repair of a DSB is reduced when Las17 is degraded but is eliminated when Las17 is inactivated in an *mre11*Δ strain [28], when apparently all resection is blocked. In metazoans, inhibiting Arp2/3 reduced the intensity of RPA foci and ssDNA formation at site‐specific DSBs. While knockdown of Mre11 in *Drosophila* lowered Arp2 association with γH2AX [43], it is unclear if Mre11‐dependent resection is required for Arp2/3 recruitment to DSBs.

An open question is how Arp2/3 facilitates resection and if this mechanism is the same for the initiation of resection. To better understand the role of Arp2/3 in resection it would be important to identify which chromatin remodelers, helicases, nucleases, and other DNA damage proteins interact with Arp2/3. Immunoprecipitation‐mass spectroscopy (IP‐MS) [55] can be used to identify proteins that are associated with Arp2/3 following a DSB. If Arp2/3 is required for the recruitment of certain proteins to DSBs for resection, then IP‐MS of proteins recruited to site‐specific DSBs ± Arp2/3 inhibition can identify which proteins require Arp2/3 activity for their recruitment to DSBs. Arp2/3 might also act to remove certain barriers to resection. Identifying the types of proteins that Arp2/3 associates with will help our understanding of the molecular mechanisms of DSB end resection.

### 1.9 Actin Polymerization and DNA Damage Repair

There is growing evidence for actin and actin‐related proteins working directly at DNA damage sites. In *Xenopus* egg extracts, it was found that Arp2/3, β‐actin, and capping protein were recruited to Mre11‐enriched DSB‐containing chromatin [52]. WASP has been shown to affect the loading of the single‐strand‐binding protein RPA onto single‐stranded DNA in mammalian cells [70] and mutations in Las17, the budding yeast homologue for WASP was shown to have hypersensitivity to the DNA damaging agents hydroxyurea (HU) and methyl methanesulfonate (MMS) [82]. Las17 mutants accumulated more Rad52 foci when exposed to HU or MMS, suggesting a deficiency in repair by HR [82]. WASP and Las17 were shown to bind to RPA [82]. In *Drosophila*, Arp2/3, myosins, and F‐actin were enriched at DSBs in heterochromatin [43]. Together this suggests that Arp2/3, Las17^WASP^, Myo3, and Myo5 work directly at DSBs during DSB repair.

In HeLa and U2OS cells, it seems that β‐actin interactions with RPA regulate DNA replication and facilitate the association of RPA with ssDNA at replication forks [101]. When the pool of free β‐actin was increased with a hyper‐depolymerizing β‐actin mutant, there was an increase in β‐actin to RPA association coupled with slower rates of replication [101]. Since WASP brings G‐actin to Arp2/3 to initiate actin polymerization and WASP has been shown to be key in the loading of RPA onto ssDNA, it is possible that WASP brings β‐actin bound RPA to ssDNA for loading. To test whether WASP‐β‐actin‐RPA form a complex, proximity ligation assays (PLA) and Western blots from immunoprecipitation of these proteins can be used to assess whether these three proteins form a complex. It is important to understand how WASP and actin modulate end‐resection and to determine how this relates to the reduced resection of DSB ends that has been reviewed here.

In addition to its role in DSB repair, Arp2/3, WASp, and N‐WASp have been shown to play roles in fork protection at stalled replication forks [101]. F‐actin was found to accumulate in the nucleus in IMR90 fibroblast cells following treatment with the replication stress‐inducing drug aphidicolin or hydroxyurea [102]. The formins DIAPH1, 2, and 3 were found to be enriched at stalled replication forks [101]. Since formins play a role in replication stress, it is possible that they have an additional role in DSB repair and resection. Bni1 and Bnr1 are the budding yeast formins that control actin cable assembly [103]. A deletion of either or both of these mutants could give insight into the role of formins in DSB repair.

### 1.10 Arp2/3 And an Alternative DNA Damage Checkpoint

The DNA damage checkpoint (DDC) is activated to halt cell cycle progression and allow cells a chance to repair the DSB before proceeding through mitosis [104, 105]. In budding yeast, activation of the DDC is mainly controlled through the effector kinase Mec1^ATR^ with a minor contribution by the Tel1^A^ kinase [106, 107, 108, 109]. Mutations in Rad50 or a deletion of Mre11 or Sae2^CtIP^ in budding yeast can activate the Tel1‐Mre11 (TM) checkpoint [110]. We found that this checkpoint was activated when Arp2/3 was inhibited before DSB induction. Yeast treated with CK‐666 before DSB induction arrest for about 4 h as opposed to 12–15 h in untreated cells [28, 111]. This arrest is dependent on both Tel1 and Mre11 demonstrating that inhibition of Arp2/3 triggers the TM checkpoint [28].

How Sgs1/DNA2 or Exo1 are loaded onto Mre11‐resected DBS ends is not known. RPA and MRX play important roles in Sgs1/DNA2 recruitment [6, 112]. In mammalian cells, EXO1 is recruited and regulated by PAR [113]. Long‐range resection is not dependent on MRX. In budding yeast, Sgs1 recruitment and function at DSBs is regulated through SUMOylation by the Smc5/6 complex [114, 115]. In HEK293T cells, SUMOylation of EXO1 results in degradation of EXO1 [116], suggesting that SUMOylation also downregulates resection. Interestingly, overexpression of Exo1 in budding yeast was able to compensate for inhibition of Arp2/3 after resection was already initiated, but Exo1 overexpression was not able to initiate resection when Arp2/3 was inhibited [28].

## 2 Conclusions

**FIGURE 3:**
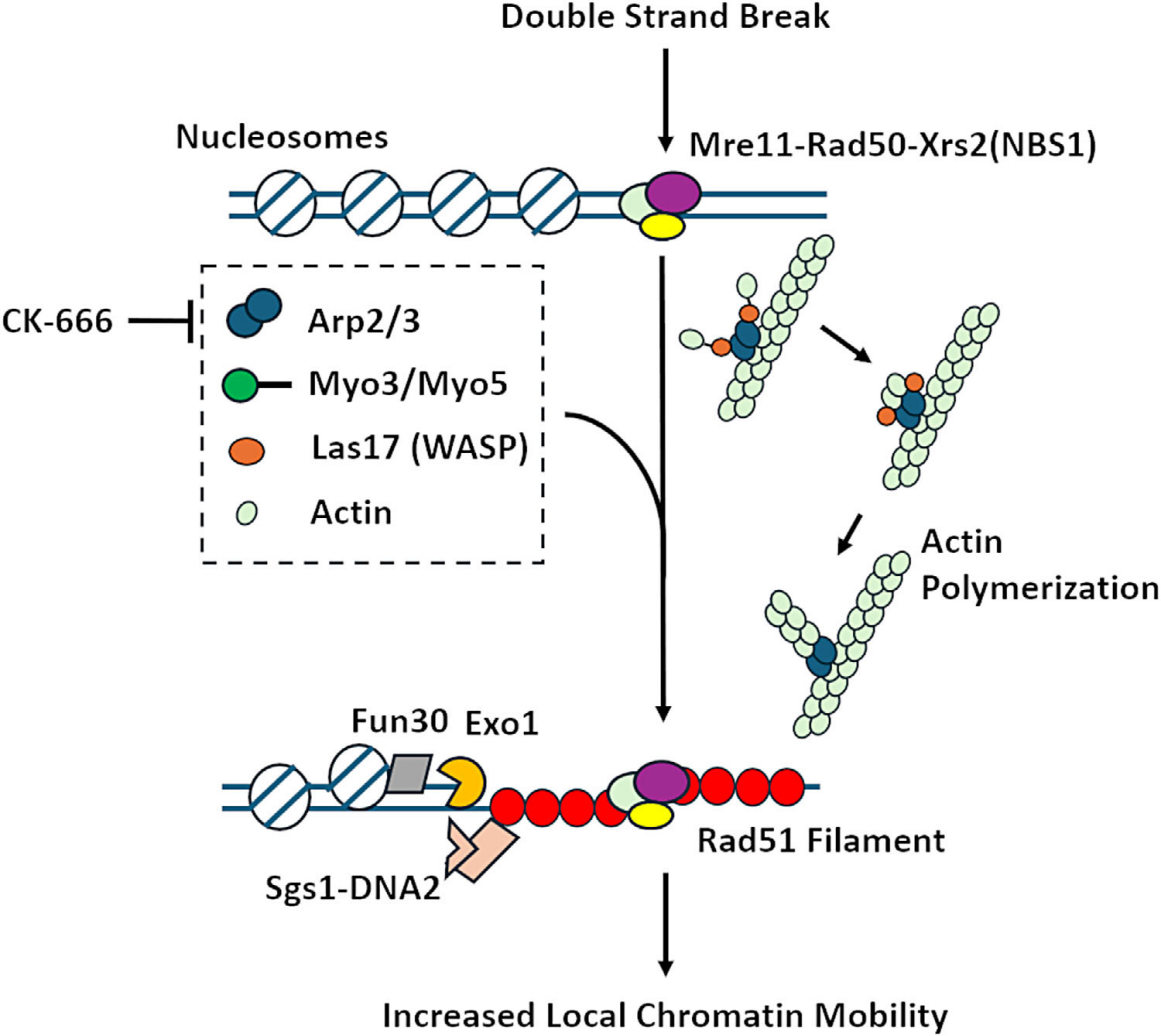
Model of Arp2/3 and resection in budding yeast. Following a DSB event the MRX^MRN^ complex is quickly recruited to the ends of the DSB. Actin nucleation through Arp2/3, Las17, and Myo3/Myo5 results in resection of dsDNA to ssDNA through Exo1 and Sgs1‐DNA2 and subsequent Rad51 filament formation. During endocytosis Las17 brings monomeric actin to Arp2/3 to nucleate new actin filaments [58]. It is possible that a similar process of Arp2/3 mediated actin nucleation occurs near DSBs. Resection of DSB ends contributes to the increased local chromatin mobility found near DSB sites. Regulation of the rate of resection, through chromatin remodelers such as Fun30, affects this increase in local chromatin mobility.

Arp2/3, myosin motor proteins, and actin polymerization have been shown to have roles in DNA damage repair processes, including DSB end resection, heterochromatic DSB relocation to the nuclear periphery, and activation of the DNA damage checkpoint (Figure 3 and Table 1); but there remain a number of open questions (see Box 1). Of note, Arp2/3 was required for clustering of DSBs in euchromatin in *Drosophila* but did not require myosins. These functions is conserved across evolution as it has been reported in budding yeast, *Xenopus* egg extracts, *Drosophila*, and in mammalian cells. While disruption of Arp2/3 or myosins through drug inhibition or deletion mutants lowered the mobility of DSBs, lower DSB mobility did not correlate with a change in repair through HR in yeast or *Drosophila*. The effects of local mobility on DSB repair through HR might be better studied when the DSB is further from the centromere as distance from the centromere has an effect on repair through HR. However, changes in resection and the length of the DNA damage checkpoint correlated with repair efficiency through HR. Arp2/3 and WASP play an evolutionarily conserved role in resection as shown in mammalian cells and budding yeast. Further work needs to be done to explain how Arp2/3 mediates resection and explore the relationship between myosins and DSB mobility. One key question about the role of Arp2/3 in DSB repair is whether Arp2/3 acts directly at the DSB sites or works indirectly to move the DSB to the nuclear periphery. Evidence for Arp2/3 and WASP acting at DSB sites can be seen in WASP loading of RPA on ssDNA in mammalian cells and Arp2 localization with γH2AX in *Drosophila*. Identifying where and when Arp2/3 is recruited in response to DSBs will give insights into how Arp2/3 fits into the DNA damage response.

**TABLE 1:** Role of Arp2/3 and WASP in DNA damage repair across species.

Species	Function
*S. cerevisiae*	DSB mobility [28] Facilitates resection [28] Myo3 and Myo5 dependent [28] DNA damage checkpoint [28]
*Drosophila*	DSB mobility [43] Relocation of heterochromatic DSBs to the nuclear periphery [43] Myo1A/1B/V, Myo5, Unc45, and Smc5/6 dependent [43]
*Mus. musculus*	DSB mobility [43] Relocation of heterochromatic DSBs to the nuclear periphery [43]
*H. sapiens*	DSB mobility [52] Facilitates resection [52] WASP mediates RPA loading [82] Mediates homologous recombination [52] Replication fork protection [48]

**Open Questions**

How does Arp2/3 and WASP promote resection on a mechanistic level?

How does inhibiting long‐range resection impair increased mobility at DSB ends?

In DSB repair, do Arp2/3, WASP, and myosin proteins act directly at DSB sites or function indirectly to promote DSB mobility?

How do changes in local and global chromatin mobility affect DSB repair efficiency through homologous recombination?

What is the relationship between different types of myosin and DSB repair?

## Author Contributions

The review was conceived and written by F.Z and J.E.H.

## Funding

Work in the Haber lab has been supported by NIH grant R35 GM127029.

## Conflicts of Interest

The authors declare no conflicts of interest.

## Data Availability

The data that support the findings of this study are available on request from the corresponding author. The data are not publicly available due to privacy or ethical restrictions.
